# *In vitro* and *ex vivo* scolicidal effects of *Olea europaea* L. to inactivate the protoscolecs during hydatid cyst surgery

**DOI:** 10.1016/j.amsu.2019.04.006

**Published:** 2019-04-24

**Authors:** Massumeh Niazi, Mojgan Saki, Maryam Sepahvand, Sareh Jahanbakhsh, Mehrdad Khatami, Mania Beyranvand

**Affiliations:** a Student Research Committee, Lorestan University of Medical Sciences, Khorramabad, Iran; b Razi Herbal Medicines Research Center, Lorestan University of Medical Sciences, Khorramabad, Iran; c Student Research Committee, Bam University of Medical Sciences, Bam, Iran; d Department of Surgery, Lorestan University of Medical Sciences, Khorramabad, Iran

**Keywords:** Cystic echinococcosis, *Echinococcus granulosus*, Protoscoleces, In vitro, Ex vivo, Olive

## Abstract

**Background:**

Nowadays, surgery is considered as one of the most important treatments for hydatidosis. Due to laceration, the cyst and spread of the content within it (protoscoleces) during the surgery that can put the patient at the risk of re-infection, anaphylaxis shock and even death, surgeons use some chemical drugs as protoscolicidal agents. The study is aimed to evaluate the scolicidal effects of olive (*Olea europaea* L.) leaf extract on hydatid cyst protoscoleces *in vitro* and *ex vivo*.

**Methods:**

After the collection of protoscoleces from sheep livers infected with fertile hydatid cysts, they were treated with various concentrations of olive leave extract (75–300 mg/mL) for 5–30 min *in vitro* and *ex vivo*. Finally, the mortality of protoscoleces was assessed by the eosin exclusion test (0.1% eosin staining).

**Results:**

The mean of the mortality of protoscoleces was 100% after 10 min of incubation with the concentration of 300 mg/ml of *O. europaea* leaves extract. On the other hand, the mean of the mortality of protoscoleces after 20 min of incubation with the concentration of 150 mg/ml of *O. europaea* leaves extract was 100%. After injection of *O. europaea* leaves extract directly into the hydatid cyst (*ex vivo*), the mean of the mortality of protoscoleces was 100% after 12 and 25 min of incubation with the concentration of 300 and 150 mg/ml of *O. europaea* leaves extract, respectively; indicating that the extract requiring a further time to display a potent protoscolicidal effects.

**Conclusion:**

Based on the findings of the study, it can be concluded that the extract of olive leaf had a significant scolicidal activity on hydatid cyst protoscoleces. However, further research, especially in human and animal subjects, are required to reach this conclusion.

## Highlights

• Scolicidal effects of olive extract on hydatid cyst protoscoleces *in vitro* and *ex vivo.*
• Protoscoleces were treated with various concentrations olive extract (75–300 μl/ml) for 5–30 min.
• 100% of protoscoleces were killed after 10 min incubation with the of 300 mg/ml.
• Ex vivo, olive extracts requiring a further time to display a potent protoscolicidal effects.
• Olive leaf may be used as a scolicidal agent in hydatid cyst surgery.

## 1 Introduction

Cystic echinococcosis (hydatidosis) is one of the most important zoonotic diseases that is caused by the larval stage of *Echinococcus granulosus,* a parasite from the family of cestodes [1]. Although the disease is reported anywhere in the world, its prevalence is higher in less developed countries, causing damage to the health and economic structures of communities [2]. Dogs and carnivores are the main host of this parasite and humans are contaminated with eating contaminated materials with parasite eggs, e.g. water, vegetables and any physical agent that can enter the parasite eggs into the host's mouth [3]. After eating eggs from the definitive host, parasites penetrate the intestines and spread through the bloodstream to the liver, lungs and, sometimes, other tissues such as the kidney, heart, thyroid, breast, etc. [3]. After replacing the larval stage in the target tissue, the cyst is formed in that limb and, depending on the location of the infection, affects the function of the organ [4]. The symptoms of the disease also rely on the affected organ, the size of the cyst and its exact location in the affected part as well as the reactions between the cyst and the affected organ [4]. Hydatid cyst treatment methods are faced with limitations; azoles are effective in the cases where cysts are small and few, but they are not responsive in all the cases and are not without unwanted and harmful effects [4].

Surgery is considered today as one of the most important treatments for hydatidosis. Due to laceration, the cyst and spread of the content within it (protoscoleces) during the surgery that can put the patient at the risk of re-infection, immunological reactions such as anaphylaxis shock and even death, surgeons use some chemical drugs such as hypertonic saline, Ag-nitrate and cetrimide to minimize surgical dangers [5]. However, recent studies have confirmed severe complications such as necrosis, fibrosis and impaired performance of liver and gallbladder following the use of these agents [6,7]. So, it is necessaryfor surgeons to try to achieve new scolicidal agents in order to maintain the human health.

Since the beginning of the creation, humans have been seeking plant remedies for treatment due to their abundance and diversity [8]. Olive, with the scientific name *Olea europaea*, is a small tree of olea genus that has wide dispersion from the Mediterranean Area, North Africa, Southeast Asia, North to South China, Scotland and East Australia to Iran. Based on the previous reviews, asthma, gallstones, hypertension, diarrhea, respiratory and urinary tract infections are the examples of diseases treated with different parts of olives in the traditional medicine [9]. However, recent studies have proven new pharmacological properties of this plant including anticancerous, antidiabetic, antihypertensive, anti-inflammatory [10,11]. Today it has been proven that *O. europaea* due to having some antimicrobial compounds such as polyphenolic, phenols and triterpenoid compounds including oleanolic acid, maslinic acid and ursolic acid revealed potent antimicrobial activities against various pathogenic bacterial, fungal and parasitic strains [10,11]. What we have been pursuing as the goal in this study is the assessment of the scolicidal effects of olive leaf extract on hydatid cyst protoscoleces both *in vitro* and *ex vivo*.

## 2 Materials and methods

### 2.1 Plant materials

In September 2017, the leaves of *O. europaea* were obtained from rural regions of Khorramabad District, Lorestan Province, located in the west of Iran. By a botanist at Razi Herbal Medicines Research Center, Lorestan University of Medical Sciences (Khorramabad, Iran), the plant materials were identified. A voucher specimen of the plant materials was deposited at the herbarium of Razi Herbal Medicines Research Center, Khorramabad, Iran (RH 1165).

### 2.2 Preparation of plant extract

About 20 g air-dried and pulverized *O. europaea* leaves were put into a cellulose cartridge and extracted in a 250 mL schott Duran Soxhlet extractor (Germany) with 200 mL ethanol: H_2_O (70:30) for 16 h. On a rotatory evaporator at 40 °C, the solvent was evaporated and, until the analysis, the residues were kept at 4 °C [[12], [13], [14]].

### 2.3 Collection of protoscoleces

With referring to slaughterhouses in Khorramabad, Iran, protoscoleces of hydatid cysts were collected from the liver of the infected sheep and goats and transferred to Parasitology Laboratory, Lorestan University of Medical Sciences, Iran. By a 50 mL syringe, the hydatid fluid was aspirated, aseptically carried into a flask and left to set for 30 min for protoscoleces to settle down. The supernatant was discarded and the protoscoleces were washed twice with PBS (pH 7.2) solution. The number of protoscoleces per mL was adjusted as 2 × 10^3^ protoscoleces in 0.9% NaCl solution with at least 90% viability rate. The viability of the protoscoleces was approved by their flame cell motility and impermeability to 0.1% eosin solution (Sigma Aldrich, St Louis, MO, USA) under a light microscope [15,16].

### 2.4 *In vitro* protoscolicidal activity

*O. europaea* leaves extract at the concentrations of 75, 150 and 300 mg/mL (0.2 ml) was added to each test tube containing 0.2 ml of protoscoleces and, then, kept for 5, 10, 20 and 30 min at 37 °C. After this time, 50 μL of 0.1% eosin stain was added to the protoscoleces. Then, the protoscoleces were smeared on a glass slide, covered with a coverslip and tested under a light microscope [17,18]. Normal saline + tween 20 and Ag-nitrate were also considered as the negative and positive controls, respectively.

### 2.5 *Ex vivo* protoscolicidal activity

In this survey, *ex vivo* protoscolicidal effects of the olive leave extract were evaluated. Briefly, more than 50% of the liquid of fertile hydatid cysts (four cysts) was aspirated and the extract at the concentrations of 75, 150 and 300 mg/mL was injected into the cysts. In the next step, an aliquot from the liquid containing the protoscoleces was collected at 7, 10, 12,15, 20, 25 and 30 min after the exposure. Subsequently and similar to the *in vitro* protoscolicidal assay, the viability of protoscoleces was determined by eosin exclusion test [19].

### 2.6 Statistical analysis

In the present study, all the tests were done in triplicate. SPSS 17.0 statistical package (SPSS Inc., Chicago, IL, USA) was used to analyze the obtained data. P-value of less 0.05 was considered statistically significant between the test and control groups.

### 2.7 Results

#### 2.7.1 In vitro protoscolicidal activity

The mean of the mortality of protoscoleces was 100% after 10 min of incubation with the concentration of 300 mg/ml of *O. europaea* leaves extract. On the other hand, the mean of the mortality of protoscoleces after 20 min of incubation with the concentration of 150 mg/ml of *O. europaea* leaves extract was 100%. The scolicidal effects of different concentrations of *O. europaea* leaves extract against the protoscoleces of hydatid cyst for 5, 10, 20 and 30 min are shown in Table 1. The mortality rate in protoscoleces in the negative and positive controls was 5.6% after 30 min and 100% after 5 min of exposure, respectively; indicating that *O. europaea* leaves extract exhibited the considerable protoscolicidal effects compared with the control group (P < 0.001).

**Table 1:** *In vitro* protoscolicidal effects of olive leave extract against protoscoleces of hydatid cyst at various concentrations following various exposure times.

Concentration (mg/ml)	Exposure time (min)	Mean of mortality rate (%)
300	5	86.6 ± 5.1
	10	100 ± 0.0
	20	100 ± 0.0
	30	100 ± 0.0
150	5	46.3 ± 3.6
	10	92.6 ± 4.15
	20	100 ± 0.0
	30	100 ± 0.0
75	5	17.0 ± .15
	10	44.3 ± 2.51
	20	86.6 ± 5.1
	30	100 ± 0.0
Normal saline + tween 20	5	0.0 ± 0.0
	10	2.6 ± 1.15
	20	4.3 ± 0.57
	30	5.6 ± 1.15
Ag-nitrate	5	85.6 ± 2.51
	10	100 ± 0.0
	20	100 ± 0.0
	30	100 ± 0.0

#### 2.7.2 Ex vivo protoscolicidal activity

After injection of *O. europaea* leaves extract directly into the hydatid cyst, the mean of the mortality of protoscoleces was 100% after 12 and 25 min of incubation with the concentration of 300 and 150 mg/ml of *O. europaea* leaves extract, respectively; indicating that the extract requiring a further time to display a potent protoscolicidal effects. The results showed that, at the concentration of 300 mg/ml and incubation time of 10 min, 100% of protoscoleces was killed within the hydatid cyst (Table 2). The mortality rate in protoscoleces in the negative and positive controls was 5.3% after 30 min and 100% after 12 min of exposure, respectively; indicating that *O. europaea* leaves extract exhibited the considerable protoscolicidal effects compared with the control group (P < 0.001).

**Table 2:** Ex vivo protoscolicidal effects of olive leave extract against protoscoleces of hydatid cyst at various concentrations following various exposure times.

Concentration (mg/mL)	Exposure time (min)	Mean of mortality rate (%)
300	7	46.6 ± 4.1
	10	71.3 ± 5.15
	12	100 ± 0.0
	15	100 ± 0.0
	20	100 ± 0.0
	25	100 ± 0.0
	30	100 ± 0.0
150	7	16.3 ± 1.15
	10	31.6 ± 1.15
	12	55.3 ± 2.15
	15	74.6 ± 3.05
	20	90.6 ± 6.1
	25	100 ± 0.0
	30	100 ± 0.0
75	7	9.3 ± 1.52
	10	18.6 ± 1.51
	12	32.6 ± 2.51
	15	57.6 ± 3.05
	20	70.3 ± 1.52
	25	83.6 ± 3.05
	30	95.0 ± 3.00
Normal saline + tween 20	7	0.0 ± 0.0
	10	0.0 ± 0.0
	12	0.0 ± 0.0
	15	2.6 ± 1.15
	20	3.00 ± 1.00
	25	4.6 ± 1.15
	30	5.3 ± 0.57
Ag-nitrate	7	85.6 ± 2.88
	10	95.0 ± 2.6
	12	100 ± 0.0
	15	100 ± 0.0
	20	100 ± 0.0
	25	100 ± 0.0
	30	100 ± 0.0

## 3 Discussion

The basic treatment of the hydatidosis in humans is the surgical procedure and removal of cysts from the body, which should be done with a special elegance, because if during the surgery, the fluid contained in the cysts or parts of it spread in the body, secondary cysts can be formed and even death of the patient might occur [4]. Sometimes due to the expanse of cysts in various organs of the body or the presence of cysts in a sensitive and dangerous area, the access to it and surgical activity is difficult, in which case medication will be used. However, the side effects of these drugs can be associated with certain risks [[4], [5], [6]].

Therefore, it is natural that humans seek to discover new scolicidal agents with minimal risks and side effects and most efficiency to minimize these problems. A good scolicidal substance should have properties like low toxicity, limited unwanted side effects, high performance and ability to maintain its properties in diluted hydatid fluid [1]. Based on what has been learned from the scientific literature, plant resources have always been considered as a good source of access to medicines due to their variety and availability [8]. In this study, since the best model for evaluation of scolicidal effects of a new agent is *in vitro* and *ex vivo* assay, we decided to investigate the effects of protoscolicidal extract of olive leaves extract in *in vitro* and *ex vivo* model. Protoscoleces were obtained from infected sheep liver exposed to concentrations 75, 150 and 300 mg/ml of extract of olive leaves. The findings of the study indicated that the mortality rate of different doses of this extract, especially at the concentrations 300 and 150 mg/ml, showed the remarkable scolicidal activity in comparison with the control group *in vitro*. *Ex vivo* assay showed that after injection of *O. europaea* leaves extract directly into the hydatid cyst, the mean of the mortality of protoscoleces was 100% after 12 and 25 min of incubation with the concentration of 300 and 150 mg/ml of *O. europaea* leaves extract, respectively; indicating that the extract requiring a further time to display a potent protoscolicidal effects.

So far, many studies have been done on the effects of scoliocidal of various herbs in hydatid cyst protoscoleces. Gholami et al. (2013) investigated the lethal effects of methanolic extract of *Sambucus ebulus;* their results showed that, at 100 mg/ml concentration and 60 min, the mortality rate of the proto-vasculitis was 98.6% [20]. In 2016, in the study by Rahimi-Esboei et al. which was performed on the ultrasonic extract of *Allium sativum*, it was found that *A. sativum* at the concentrations of 50 and 100 mg/ml after 180 min of exposure killed 86% and 98% of protoscoleces, respectively [21]. In the study of Moazeni et al. (2011), the mortality rate of protoscoleces was 100% when they were exposed to 100 mg/ml of *Zingiber officinale* for 30 min period [22]. Galehdar et al. (2018) in a report put forward the results of investigating the effects of *Nectarscordum koelzi* extract on hydatid cyst protoscoleces. The finding of this study showed that the extract at the concentrations of 250 and 500 mg/ml, after 10 and 20 min, killed all the protoscolexes, respectively [23]. In addition, reviews have shown the protoscolicidal effects of a number of plant [8]. The results of several studies by Mahmoudvand et al. have represented the scolicidal activities of plants such as *Nigella sativa, Pistacia vera, Pistacia khinjuk, Pistacia atlantica, Bunium persicum* and *Myrtus cumminus* [16,18,24,25].

However, recent studies have proven new pharmacological properties of *O. europaea* including anticancerous, antidiabetic, antihypertensive, antiinflammatory and antimicrobial effects [10]. Based on the past studies, polyphenolic, phenols and triterpenoid compounds including oleanolic acid, maslinic acid and ursolic acid are considered as the main components of *O. europaea* [10]. Reviews have reported the anti-parasitic activity of these components [[26], [27], [28]]. Considering the antimicrobial mechanisms, studies demonstrated that **these** compounds through damaging the cytoplasmic membrane, disrupting cell peptidoglycans, as well as interference with the production procedures of certain amino acids necessary for the growth of microorganisms can showed their antimicrobial mechanisms [[29], [30], [31], [32]]. Therefore, we can suggest the protoscolicidal activity of *O. europaea* due to having such constituents.

## 4 Conclusion

Based on the findings of the study, it can be concluded that the extract of olive leaf has a significant scolicidal activity on hydatid cyst protoscoleces. However, further study, especially in human and animal subjects, are required to reach this conclusion.

## Conflicts of interest

The authors declare no conflict of interest in this study.

## Ethical approval

No need.

## Sources of funding

No.

## Author contribution

Massumeh Niazi: study design, data collection.

Maryam Sepahvand: data analysis.

Mojgan Saki: critical review.

Sareh Jahanbakhsh: data collection.

Mehrdad Khatami: data analysis.

Mania Beyranvand: writing, supervisor.

## Conflicts of interest

No.

## Research registration number

–

## Guarantor

Massumeh Niazi.

## Provenance and peer review

Not commissioned, externally peer reviewed.
